# Cancer Attributable to Asbestos Exposure in Shipbreaking Workers: A Matched-Cohort Study

**DOI:** 10.1371/journal.pone.0133128

**Published:** 2015-07-20

**Authors:** Wei-Te Wu, Yu-Jen Lin, Chung-Yi Li, Perng-Jy Tsai, Chun-Yuh Yang, Saou-Hsing Liou, Trong-Neng Wu

**Affiliations:** 1 National Institute of Environmental Health Sciences, National Health Research Institutes, Miaoli, Taiwan; 2 Institute of Occupational Medicine and Industrial Hygiene, National Taiwan University, Taipei, Taiwan; 3 Department of Public Health, College of Medicine, National Cheng Kung University, Tainan, Taiwan; 4 Department of Environmental and Occupational Health, College of Medicine, National Cheng Kung University, Tainan, Taiwan; 5 Institute of Public Health, Kaohsiung Medical University, Kaohsiung, Taiwan; 6 Department of Nursing, HungKuang University, Taichung, Taiwan; University of Montana, UNITED STATES

## Abstract

**Purpose:**

Long-term follow-up studies of asbestos-related cancer in shipbreaking workers are lacking. This study examines the relationship between cancer incidence and asbestos exposure among former Taiwan shipbreaking workers.

**Methods:**

A total of 4,427 shipbreaking workers and 22,135 population-based matched controls were successfully followed in this study. The study cohort was linked to the Taiwan Cancer Registry for new cancer cases. The adjusted hazard ratio (aHR) for cancer was calculated for the shipbreaking workers with Total Exposure Potential Scores (TEP) for asbestos.

**Results:**

Follow-up generated 109,932 person-years, with 940 deaths and 436 cancer cases, among 4,427 shipbreaking workers from 1985 to 2008. The high asbestos exposure group also had a statistically significant increase in the risk of overall cancer (aHR= 1.71; 95% CI: 1.42-2.05), esophagus cancer (aHR= 2.31; 95% CI: 1.00-5.41), liver and intrahepatic bile duct cancer (aHR= 1.60; 95% CI: 1.08-2.36), and trachea, bronchus, and lung cancer (aHR= 3.08; 95% CI: 1.80-5.25). Mesothelioma cases were found in the high asbestos exposure group. Moreover, overall cancer, esophagus cancer, and trachea, bronchus, and lung cancer were seen in a dose-dependent relationship with asbestos exposure.

**Conclusions:**

This study presented the elevated trend of asbestos exposure with cancer incidence for overall cancer, esophagus cancer, and trachea, bronchus, and lung cancer among shipbreaking workers. Those workers previously exposed to asbestos should receive persistent monitoring in order to early detect adverse health outcomes.

## Introduction

Asbestos exposure and its health risks still are concerned to the scientific and technological community, policymakers and the general public. Although all forms of asbestos are banned in 52 countries [1], there remain approximately 125 million people around the world who are now exposed to asbestos in their work environments [2]. Recycling ships for scrap that caused the asbestos hazard is a persistent and international problem. Every year, out of about 1000 ocean-going ships sold for recycling, 80% end up on the beaches of South Asia such as Bangladesh, Pakistan, India and China [3, 4]. Additionally, the ship-recycling market hit a 13-year high in 2009 and has attracted many international organizations concerned with occupational health effects from asbestos exposure [3, 4]. However, the detailed information of incidence of asbestos-related disease and cancers among shipbreaking workers are lacking, despite the fact that asbestos induced mesothelioma or lung cancer were classified as occupational disease several decades ago.

A growing amount of scientific evidence suggests that all forms of asbestos cause lung and laryngeal cancers, malignant mesothelioma, and may cause ovarian, gastrointestinal cancers [2, 5, 6]. Studies of shipbreaking or shipyard workers potentially exposed to asbestos showed a persistent increased mortality for lung cancer [7–10]. A retrospective study of shipyard Coast Guard workers reported Standardized Mortality Ratio (SMR) of 1.26 for lung cancer and 5.07 for mesothelioma [9]. Another cohort mortality study in Genoa shipyard workers also observed significantly increased SMR of 1.77 for lung cancer [8]. Meanwhile, our previous studies showed that an elevated mortality of lung cancer and overall cancers were observed in shipbreaking workers in comparison with general population [7]. Although cancer mortality has been widely accepted as the important measure of progress against cancer, it has been considered subject to more distortion than incidence. Further, up to a third of cancer cases will not die of cancer, and cancer mortality statistics do not report their experience at all [11].

Taiwan was the largest shipbreaking nation in the world with about 65% of the waste ships being crushed there, and it accounted for more than 67 million gross tonnage (GT) and were scrapped from 1977 to 1988 in Taiwan (Table 1) [3]. These past experiences in Taiwan provided a unique opportunity to study the long-term effects of the shipbreaking workers on the development of asbestos-related cancer. Therefore, a matched-cohort study was conducted to link shipbreaking workers with the Taiwan Cancer Registry (TCR). The current study was to examine the hypothesis that shipbreaking workers have experienced an increased risk of neoplasm, particularly for asbestos-related cancers.

**Table 1:** The Gross Tonnage of obsolete ships in Taiwan, 1977–1992.

Year	1000 Gross Tonnage	T_k_/T_max_ ^a.^
1977	3,391	0.433
1978	6,042	0.772
1979	3,969	0.507
1980	4,409	0.563
1981	5,268	0.673
1982	7,829	1.000
1983	7,815	0.998
1984	6,687	0.854
1985	7,822	0.999
1986	7,773	0.993
1987	4,415	0.564
1988	1,521	0.194
1989	164	0.021
1990	2	0.0003
1991	48	0.006
1992	83	0.011
**Total**	**67,238**	

^a.^ Using the maximum number of GT of ships broken in 1982 as the denominator.

## Methods

### Study population

This study adopted a retrospective matched-cohort study design. The cohort subjects were members of the 1985 Kaohsiung Shipbreaking Workers Union who participated in the state-run Labor Insurance Program. A total of 70% of workers employed in the shipbreaking industry were members of the Union in order to be covered by insurance during this period. These workers had been employed for over a year and the date of their first employment in the shipbreaking industry had begun in 1975. The whole cohort, between 1975 and 1989, comprised 4,962 workers. By the end of December of 2008, the researchers excluded 2 shipbreaking workers who were diagnosed with cancer before employment and 533 shipbreaking workers who were not enrolled in the Taiwan National Health Insurance Program. Thus this study followed 4427 subjects in shipbreaking cohort, and a follow-up rate was 89.2%. Then 4427 shipbreaking workers were linked Registry for Beneficiaries, and each shipbreaking workers was matched to five general population control, using the same age, sex and living area as matching factors. Finally, a total of 4427 shipbreaking workers and 22135 population-based matched cohort were linked with the Taiwan Cancer Registry (TCR) to find new cases of cancer. Data analyzed in this analysis were administrative data, and all personal identification numbers were scrambled. This study was approved form the National Health Research Institutes (NHRI) Institutional Review Board (IRB), Miaoli County, Taiwan, approved, and allowed us to dispense with informed consent.

### Data sources for outcomes

In the present study, the deterministic record linkage strategy was used to pick Personal Identification Numbers (PIN) for the study subjects as their unique identifiers. Records that share the same value identified the same person. Information on new cases of cancer was obtained from the TCR, which was set up in 1979 to survey the mortality rates and the incidence of cancer. Under the current system, the TCR catch 97% of cancer cases in Taiwan [12]. The percentage of Morphologically Verified Cases (MV %) and the percentage of Death Certificate Only Cases (DCO %) help point if the cancer registry is of good quality. High data quality represented by a MV% would be 100% and DCO% would be 0% [13]. The DCO% of the cancer in the TCR decreased from 8.78% in 1998 to 0.85% in 2010. The MV% ranged from 87.5% in 2002 to 91.11% in 2010 [12]. It shows that the quality of the TCR is similar to other well-established cancer registries in the world [13, 14]. The cancer found in participant records was coded based on the *International Classification of Diseases for Oncology*, *Third Edition (ICD-O3)*.

### Exposure assessment for asbestos

A panel of seven experts was asked to assess exposure subjectively. The panel consisted of two occupational hygienists, four occupational physicians, and a risk assessment expert. The occupational hygienists previously worked in government agencies and had experience in the field of asbestos exposure assessment in the shipbreaking workplace. Occupational physicians are epidemiologists who also have had experience with the treatment of patients with a history of asbestos exposure. The experts responded to a survey with a 0-to-10 point rating scale (0: mild to 10: serious). The purpose was to score the Exposure Intensity (EI) for asbestos according to the eight job titles in the shipbreaking industry consists of flame cutters, odd-jobbers, lifters, supervisors, knockers, sorters, drivers, and administrators. The average amount of EI for asbestos within the eight job titles was: flame cutters (scores = 9.6), odd-jobbers (7.1), lifters (5.7), supervisors (5.6), knockers (4.6), sorters (6.0), drivers (2.6), and administrators (2.1). Only one workplace survey by Dr. Yi-Chang Lin in 1987 was conducted eight-hour full-shift samplings upwind and downwind from the 91 areas of Kaohsiung harbor, and he used transmission-electronic microscopy (10,000x) and phase-contrast microscopy (450 x) to determine asbestos concentrations. The real amount of asbestos within the eight job titles was: flame cutters (0.196 ± 0.202 f/cm^3^), odd-jobbers (0.208 ± 0.097 f/cm^3^), lifters (0.138 ± 0.156 f/cm^3^), supervisors (0.020 f/cm^3^), knockers (0.050 ± 0.014 f/cm^3^), sorters (0.060 f/cm^3^), drivers (0.020 f/cm^3^), and administrators (0.020 f/cm^3^). Moreover, this study assessed whether the EI for asbestos within the eight job titles reflected the real amount of asbestos to which workers in the shipbreaking workplace were being exposed. The results showed that greater amounts of asbestos in the shipbreaking workplace were highly correlated with greater exposure intensities (R = 0.878, p<0.001), particularly for Amosite and Crocidolite. The researchers also used intraclass correlation coefficient (ICC) between the raters in experts for ranking eight job titles for the EI for asbestos (n = 7; ICC = 0.890). The detailed information of the summary of the correlation between environmental asbestos levels and the EI for asbestos are presented in our previous study [15].

The study calculated the Exposure Potential Scores (EP) for asbestos based on the formula listed below [1]. Table 1 showed that the GT of ships broken in Taiwan from 1977 to 1992. The maximum number of GT of ships broken was in 1982. The Total Exposure Potential Scores (TEP) for asbestos were calculated for the eight job titles according to years of employment for each shipbreaking worker [2]. Within the TEP for asbestos subjects were categorized into high (≥ 45.46 TEP), medium (32.86–45.45 TEP), and low (< 32.86 TEP) asbestos exposure subgroups.

$$EP_{k}=EI\left(\frac{T_{k}}{T_{\text{max}}}\right)$$ (1)

Where EP: Exposure potential scores for asbestos

EI: Intensity of asbestos exposure

T_k_: The GT of ships broken in *k* ^th^ year

T_max_: The GT of ships broken in 1982 (the maximum number of GT of ships broken over the years)

$$TEP=\sum_{k=1}^{n}EP_{k}$$ (2)

Where *TEP*: The total exposure potential scores for asbestos

*k*: The *k* ^th^ year on shipbreaking job

### Statistical analysis

Those workers who survived between the time periods of when their employment began to December 31, 2008 contributed to the person-years time. Those known to have deceased before the cutoff date contributed the person-year time between their work start date and their date of death or when they were diagnosed with cancer. Cox proportional hazards model was performed to estimate the hazard ratio (HR) for various types of cancers among shipbreaking workers by each job group category (flame cutters, lifter, odd-jobbers, supervisors, and others) and by each of the asbestos exposure groups (high, medium, and low). Due to the Taiwan’s Personal Information Protection Act that was established on October 1 2012 there are new restrictions that require researchers to use symbols to express cancer cases that are less than 2 in a subgroup analysis. The analysis was used SAS software (SAS Institute, version 9.3).

## Results

By the end of the follow-up period, which generated 109,932 person-years among 4427 shipbreaking workers (Table 2). Among shipbreaking cohort, there were 940 deaths and 436 cancer cases in 24-year follow-up. The shipbreaking workers had a significant percentage difference of cancer in comparison with matched-cohort (9.9 vs. 6.7%; p<0.001). According to the categories of TEP for asbestos, workers were divided into low (n = 1525, 34.4%), medium (n = 1378, 31.1%), and high (n = 1524, 34.4%) asbestos exposure groups. Mean age of their first employment was 31.2 ± 9.1 years old and the mean age at leaving this job was 38.1 ± 9.1 years old.

**Table 2:** Demographic characteristic of the shipbreaking cohort.

Demographic characteristic	Cohort (N = 4427, person-years = 109932.4)		Control (N = 22135, person-years = 588286.5)		
	*n*	*(%)*	*n*	*(%)*	*p-value*
Vitality status					<0.001
Alive	3487	(78.8)	20254	(91.5)	
Deceased	940	(21.2)	1881	(8.5)	
Cancer status					<0.001
Cancer group	436	(9.9)	1488	(6.7)	
Non-cancer group	3991	(90.1)	20647	(93.3)	
Sex					1.000
Men	3732	(84.3)	18660	(84.3)	
Women	695	(15.7)	3475	(15.7)	
Year of birth					
≤ 1945	1149	(26.0)	5755	(26.0)	0.993
1945~1955	1890	(42.7)	9429	(42.7)	
≥ 1956	1388	(31.3)	6951	(31.3)	
Place-of-residence					1.000
North area	241	(5.4)	1205	(5.4)	
Central area	131	(3.0)	655	(3.0)	
South area	4030	(91.0)	20150	(91.0)	
East area	25	(0.6)	125	(0.6)	
Asbestos exposure groups					
Low (< 32.86 TEP)	1525	(34.4)			
Medium (32.86–45.45 TEP)	1378	(31.1)			
High (≥ 45.46 TEP)	1524	(34.4)			
	*mean*	*(SD)*			
Age at entry workplace (years)	31.2	(9.1)			
Age at exit workplace (years)	38.1	(9.1)			
Exposure duration (years)	6.9	(1.8)			

Average age of cancer diagnosis and adjusted hazard ratio (aHR) were presented in Table 3, where the matched-cohort used as a reference group. A statistically significant increase in aHR was noted for overall cancer (aHR = 1.63; 95% CI: 1.46–1.81), oral cavity cancer (aHR = 2.47; 95% CI: 1.89–3.21), nasopharynx cancer (aHR = 2.18; 95% CI: 1.39–3.42), liver and intrahepatic bile duct cancer (aHR = 1.50; 95% CI: 1.16–1.94), trachea, bronchus, and lung cancer (aHR = 2.71; 95% CI: 1.99–3.69), and unknown primary cancer (aHR = 4.18; 95% CI: 1.66–10.5). Moreover, the shipbreaking workers had a statistically significant lower age of cancer diagnosis for overall cancer (54.5 ± 10.2 vs. 57.0 ± 10.9; p<0.001), liver and intrahepatic bile duct cancer (53.1 ± 8.9 vs. 56.8 ± 8.7; p = 0.001), and trachea, bronchus, and lung cancer (59.7 ± 9.0 vs. 63.6 ± 10.1; p = 0.011) in comparison with matched-cohort.

**Table 3:** Age of cancer diagnosis and hazard ratio (HR) for various types of cancers among shipbreaking workers compared with the control group.

	Cohort (n = 4427)			Control (n = 22135)							
Cancer site (ICD-O-3)	Age of cancer diagnosis			Age of cancer diagnosis							
	*n*	*mean*	*SD*	*n*	*mean*	*SD*	*p-value*	*HR* ^*a*.^	*95%CI*		
**All cancer**	**436**	**54.5**	**10.2**	**1488**	**57.0**	**10.9**	**<0.001**	**1.63**	**1.46**	-	**1.81**
Oral cavity (C00, C01, C02, C03, C04, C05, C06, C09, C10, C12, C13, C14)	82	52.3	8.5	191	53.7	8.5	0.237	**2.47**	**1.89**	-	**3.21**
Nasopharynx (C11)	27	45.8	9.9	74	48.9	9.1	0.145	**2.18**	**1.39**	-	**3.42**
Esophagus (C15)	**16**	**51.8**	**6.9**	**50**	**57.1**	**8.2**	**0.024**	1.70	0.96	-	3.01
Stomach (C16)	19	57.4	11	68	56.9	11.0	0.871	1.62	0.97	-	2.71
Colon (C18)	20	57.0	9.8	89	58.2	11.3	0.664	1.32	0.80	-	2.15
Rectum (C19, C20, C21)	12	61.3	7.6	82	57.3	11.4	0.242	0.85	0.46	-	1.57
Liver and intrahepatic bile ducts (C22)	**75**	**53.1**	**8.9**	**274**	**56.8**	**8.7**	**0.001**	**1.50**	**1.16**	-	**1.94**
Larynx (C32)	3	52.0	10.6	21	59.1	9.7	0.250	0.80	0.24	-	2.69
Trachea, bronchus, and lung (C33, C34)	**61**	**59.7**	**9.0**	**131**	**63.6**	**10.1**	**0.011**	**2.71**	**1.99**	-	**3.69**
Mesotheliomas (C35)	#	36.2	-	#	48.9	-	-	8.55	0.53	-	138.9
Thymus, heart and mediastium (C37, C38, C39, C383, C384, C388)	3	53.5	8.2	6	53.2	12.5	0.974	3.75	0.90	-	15.7
Skin (C44)	14	57.6	12.5	57	57.6	13.7	0.999	1.50	0.83	-	2.73
Prostate gland (C61)	9	66.3	3.6	66	69.1	8.8	0.088	0.82	0.41	-	1.66
Bladder (C67)	12	58.6	10.1	64	57.7	10.8	0.793	1.04	0.56	-	1.95
Kidney (C64)	4	54.9	5.7	19	59.8	9.1	0.315	1.28	0.43	-	3.85
Other urinary organs (C65, C66, C68)	5	67.3	5.2	23	64.4	8.8	0.493	1.12	0.42	-	2.96
Brain (C71)	**3**	**40.4**	**10.5**	**10**	**53.1**	**8.2**	**0.047**	2.05	0.54	-	7.81
Thyroid gland (C73)	5	52.4	8.4	26	51.8	9.1	0.901	1.09	0.41	-	2.86
Unknown primary (C80)	8	55.8	13.9	11	60.6	10.7	0.404	**4.18**	**1.66**	-	**10.5**
Leukemia (C42, C77)	8	56.5	11.1	39	56.1	13.0	0.936	1.17	0.54	-	2.53

^a.^ Adjusted for premium ratable wage per month;

Bold italic, statistically significant;

#The number of cancer cases less than 2.

Cox Proportional Hazards Model was fit to determine aHR of cancer incidence in relation to the major job titles that compared with each matched-cohort group after adjusting for premium retable wage (Table 4). For flame cutters (n = 2609), we observed a statistically significant increase of aHR for overall cancer, oral, nasopharyngeal, esophagus, stomach, liver and intrahepatic bile duct cancer, trachea, bronchus, and lung cancer, and thymus, heart and mediastium cancer. The lifters (n = 758) experienced increased aHR for overall cancer, oral cancer, and trachea, bronchus, and lung cancer. Additionally, odd-jobbers (n = 866) showed increased aHR for overall cancer, oral cancer, and colon cancer. Supervisors (n = 42) and others (n = 152) were associated with increased aHR for overall cancer, and trachea, bronchus, and lung cancer.

**Table 4:** The hazard ratio (HR) for various cancers among shipbreaking workers with different job titles, 1985–2008 ^a^.

Cancer site (ICD-O-3)		Flame cutters (n = 2609)		Lifters (n = 758)		Odd-jobbers (n = 866)		Supervisors (n = 42)		Others (n = 152)
	*N*	*HR (95% CI)*	*N*	*HR (95% CI)*	*N*	*HR (95% CI)*	*N*	*HR (95% CI)*	*N*	*HR (95% CI)*
**All cancer**	**241**	**1.68(1.45–1.94)**	**76**	**1.67 (1.29–2.16)**	**89**	**1.46 (1.16–1.85)**	**7**	**2.81 (1.14–6.95)**	**23**	**1.79 (1.11–2.87)**
Oral cavity	**50**	**2.40 (1.71–3.36)**	**21**	**2.93 (1.72–5.01)**	6	2.10 (0.83–5.33)	#	2.26 (0.22–22.9)	4	2.13 (0.66–6.88)
Nasopharynx	**19**	**2.24 (1.30–3.86)**	3	1.37 (0.38–4.86)	3	2.20 (0.57–8.45)		-	#	4.34 (0.61–30.8)
Esophagus	**13**	**1.91 (1.00–3.64)**	3	1.68 (0.45–6.21)		-		-		-
Stomach	**15**	**2.01 (1.10–3.66)**	3	1.63 (0.44–6.02)	#	0.65 (0.08–5.20)		-		-
Colon	6	0.72 (0.31–1.70)	4	1.18 (0.40–3.51)	**8**	**3.07 (1.29–7.29)**	#	10.92 (0.44–272.3)	#	1.21 (0.14–10.6)
Liver and intrahepatic bile ducts	**53**	**1.55 (1.14–2.12)**	8	0.99 (0.47–2.11)	9	1.57 (0.75–3.30)	#	8.67 (0.40–190.4)	4	1.51 (0.50–4.56)
Trachea, bronchus, and lung	**31**	**3.15 (2.01–4.92)**	**12**	**2.79 (1.38–5.65)**	10	1.47 (0.73–2.95)	#	**11.12 (1.18–105.2)**	**6**	**5.48 (1.76–17.1)**
Mesotheliomas	#	8.98 (0.55–145)								
Thymus, heart and mediastium	#	**8.46 (1.14–62.6)**								

^a.^ Adjusted for premium ratable wage per month;

Bold italic, statistically significant;

#The number of cancer cases less than 2.

**Fig 1:**
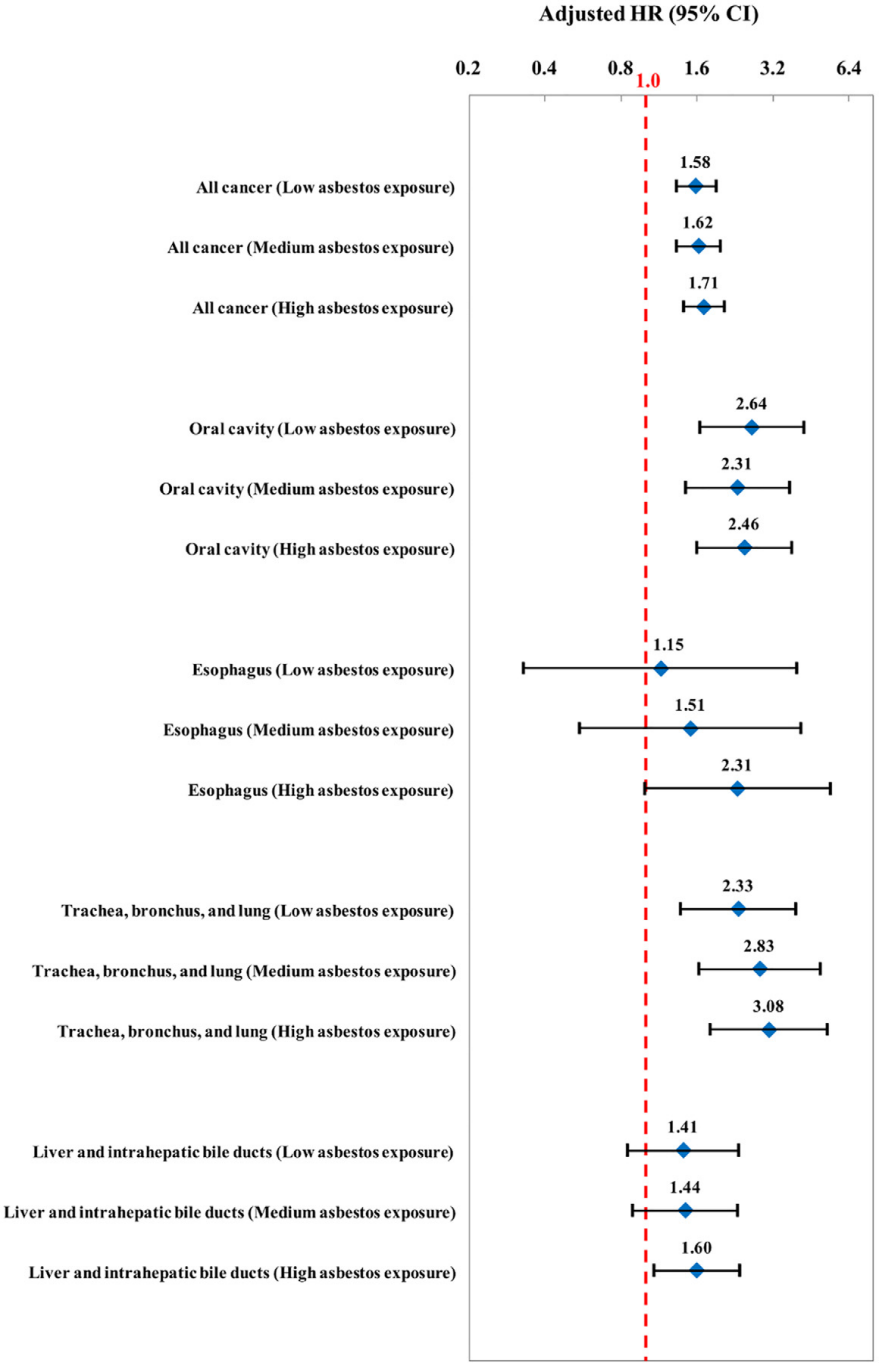
Cox model was fit to determine adjusted HR of cancer incidence in relation to TEP for asbestos in comparison with each matched-cohort group after adjusting for premium retable wage

Fig 1 showed the Cox Proportional Hazards Model used to observe the aHR for overall and cause-specific cancer among shipbreaking workers with three asbestos exposure groups. Statistically significant increase in aHR were found for overall cancer, oral cancer, and trachea, bronchus, and lung cancer in all three asbestos exposure groups. The high asbestos exposure group also had a statistically significant increase in the risk of overall cancer (aHR = 1.71; 95% CI: 1.42–2.05), esophagus cancer (aHR = 2.31; 95% CI: 1.00–5.41), liver and intrahepatic bile duct cancer (aHR = 1.60; 95% CI: 1.08–2.36), trachea, bronchus, and lung cancer (aHR = 3.08; 95% CI: 1.80–5.25), and thymus, heart and mediastium cancer (aHR = 8.83; 95% CI: 1.19–65.7). Moreover, overall cancer, esophagus cancer, and trachea, bronchus, and lung cancer were seen in excess risk and in a dose-dependent manner with asbestos expose group.

## Discussion

A retrospective matched-cohort study design, the 24-year follow-up period, and low rates of loss to follow-up of cohort subjects were the strengths of this study. The major findings of this study presented the clearer exposure-response trend of asbestos exposure with elevated cancer incidence for overall cancer, esophagus cancer, and lung cancer among shipbreaking workers. Moreover, two mesothelioma cases were found in both flame cutters and the high asbestos exposure group.

Over the 24-year follow-up period, we found 61 cases of lung cancer (ICD-O3 code: C33 and C34) and two mesothelioma cases (ICD-O3 code: C35) in shipbreaking workers. Asbestos-containing materials are commonly used for thermal insulation and surfacing materials in vessels built in the 1960s and 1970s. Amosite was found to be the dominant navy insulation fiber that amounted to approximately 86% of all the asbestos fiber in the insulation systems of an average ship [16]. Based on a report from the the International Agency for Research on Cancer, chrysotile, amosite, and canthophyllite asbestos, and mixtures containing crocidolite exposure confirms increased risks of lung cancer and mesothelioma [6]. Therefore, this is likely a major contributor to the large amount lung cancer and mesothelioma. Moreover, a previous study reported that shipyard workers were at a higher risk of mortality from lung cancer by a magnitude of 26% [9]. Similar findings in shipyard workers were also reported in Finnish (18%) and Norwegian (69%) studies [17, 18].

Elevated incidence and gradient with asbestos exposure level of overall cancer, esophagus cancer, and liver and intrahepatic bile duct cancer among shipbreaking employees were found. Findings linking an increased risk of esophagus cancer to asbestos exposure are contrasting, and there was no consistency in literature [6]. Some cohort studies reported elevated SMR for esophagus cancer in asbestos workers, based on more than 5,000 asbestos insulation boards workers in the east end of London, 3,072 asbestos textile workers in South Carolina, and 17,800 asbestos insulations workers across the USA and Canada, respectively [19–21]. Others showed negative or inconclusive correlations of esophagus cancer in a cohort of 3211 male asbestos textile workers in the United Kingdom [22], and in a cohort study of 6943 asbestos miners from Western Australia [23]. Additionally, increased mortality with an increased risk for overall cancer (SMR = 1.44) and cancer of the liver (SMR = 1.86) were observed in Genoa shipyard workers [8]. Our previous study also showed that a statistically significant increased mortality of overall cancer and liver cancer among men shipbreaking workers [7]. However, two studies of shipyard workers showed the opposite results that no elevated risk for overall cancer and cancer of the liver were found [9, 24]. This inconsistent results of cancer research in asbestos workers is possible related to latency from the onset of occupational exposure to asbestos [21].

The researchers found that those who had the occupation of flame cutter were associated with the risks of developing several kinds of cancers, and also found mesothelioma cases. Flame cutters are the most skilled and best paid workers in the shipbreaking industry, but they also have the greatest likelihood of being exposed to asbestos. Additionally, supervisors were the highest incidence of overall cancer and trachea, bronchus, and lung cancer in all job titles. This might be explained by the fact that the sample size is small in this group. However, supervisors were the most experienced and senior of the workers. They had a high of 85 percent of workers that were first employed from 1975 to 1979, and they had a longer duration of employment (8.6±1.1 years) than the other job titles. It is also possible that long-term exposure history is responsible for the increase of overall cancer found in supervisors.

To the best of the authors’ knowledge, workers from the shipbreaking industry are usually not tightly organized and tend to have a high turnover rate. Over the past decade, there have been only a few investigations of the health effects of shipbreaking workers [7, 25]. This study is based on a large cohort with a satisfactory response rate and a long follow-up period. A nearly complete follow-up rate (89.2%) greatly reduces the potential of selection bias. Additionally, our study used the same dataset to retrieve the cancer incidence information for both the shipbreaking workers and the reference population of Taiwan. This ensures good comparability in the results and a lesser likelihood of information bias. Moreover, investigation of cancer incidence gives an indication of trends in new cases, and unlike mortality trends represent dying in any one year that may have been diagnosed and treated many years earlier.

There are some limitations in this study. Due to the unavailability of lifestyle data there was a lack of information on smoking, alcohol, and diet, etc. Although this study did not get the information of smoking history in our study subjects, the smoking prevalence in Taiwan male general population (55–60%, 1976–1996) was more prevalent than male workers (48%, 1984–1997) [26, 27]. These studies might have implied that smoking might not completely explain the cancer increased noted in this study. Moreover, we are not entirely exclude the probability of potentiate effects by alcohol intake, even an obvious exposure-response relationship with asbestos exposure level of esophagus cancer and liver and intrahepatic bile duct cancer were found. Meanwhile, the lack of obvious trend for other alcohol-related cancers (ex. oral cancer, larynx cancer, and breast cancer) preclude the identification of alcohol intake as responsible for the observed excess incidence of liver cancer and esophagus cancer. Second, this historical occupational cohort study is the lack of the exposure data available in past records. In Taiwan, shipbreaking industries have regulated since 1983 and became totally prohibited in 1993, and historical exposure data in the shipbreaking workplace were unachievable. Therefore, this study used TEP for asbestos that considered working years to represent the long-term exposure levels. Third, the current study does not have information about whether the workers changed jobs during their exposure period. According to previous visits and reports about shipbreaking industries, flame cutters have the most severe exposure to hazardous substances and higher wage than others. If other shipbreaking workers changed jobs for higher wages rather than being flame cutters, it could cause a reduced amount of exposure in the assessment and underestimate the results. Fourth, it is difficult to use shipbreaking workers from the same workplace as an internal comparison group because those workers is easily exposed to asbestos. The general population of the same age, gender, and residing area with shipbreaking workers were taken as the external comparison group, but it still has some disadvantages. It supposes that a low proportion of the general population is exposed to asbestos, otherwise the existence of asbestos exposure will underestimate the results [28]. Additionally, workers tend to be healthier than the general population. The healthy worker effect may decrease the incidence of cancer and underestimate the effect of occupational exposure because of the natural higher mortality rate than the general population [29].

## Conclusion

This study confirmed increased incidence of overall cancer, esophagus cancer, and trachea, bronchus, and lung cancer which was associated with the level of exposure to asbestos among shipbreaking workers. Despite its well-documented dangers, asbestos has yet to be banned in many parts of the world. Our study results support the notation that legislation should be passed to ban asbestos-containing products not only in occupational but also in general environmental settings. Additionally, those workers previously exposed to asbestos should receive a persistent monitoring in order to early detect the adverse health outcome from exposure to asbestos.
